# A statistical virtual patient population for the glucoregulatory system in type 1 diabetes with integrated exercise model

**DOI:** 10.1371/journal.pone.0217301

**Published:** 2019-07-25

**Authors:** Navid Resalat, Joseph El Youssef, Nichole Tyler, Jessica Castle, Peter G. Jacobs

**Affiliations:** 1 Department of Biomedical Engineering, Oregon Health and Science University, Portland, Oregon, United States of America; 2 Harold Schnitzer Diabetes Health Center, Oregon Health and Science University, Portland, Oregon, United States of America; National Research Council (CNR), ITALY

## Abstract

**Purpose:**

We introduce two validated single (SH) and dual hormone (DH) mathematical models that represent an in-silico virtual patient population (VPP) for type 1 diabetes (T1D). The VPP can be used to evaluate automated insulin and glucagon delivery algorithms, so-called artificial pancreas (AP) algorithms that are currently being used to help people with T1D better manage their glucose levels. We present validation results comparing these virtual patients with true clinical patients undergoing AP control and demonstrate that the virtual patients behave similarly to people with T1D.

**Methods:**

A single hormone virtual patient population (SH-VPP) was created that is comprised of eight differential equations that describe insulin kinetics, insulin dynamics and carbohydrate absorption. The parameters in this model that represent insulin sensitivity were statistically sampled from a normal distribution to create a population of virtual patients with different levels of insulin sensitivity. A dual hormone virtual patient population (DH-VPP) extended this SH-VPP by incorporating additional equations to represent glucagon kinetics and glucagon dynamics. The DH-VPP is comprised of thirteen differential equations and a parameter representing glucagon sensitivity, which was statistically sampled from a normal distribution to create virtual patients with different levels of glucagon sensitivity. We evaluated the SH-VPP and DH-VPP on a clinical data set of 20 people with T1D who participated in a 3.5-day outpatient AP study. Twenty virtual patients were matched with the 20 clinical patients by total daily insulin requirements and body weight. The identical meals given during the AP study were given to the virtual patients and the identical AP control algorithm that was used to control the glucose of the virtual patients was used on the clinical patients. We compared percent time in target range (70–180 mg/dL), time in hypoglycemia (<70 mg/dL) and time in hyperglycemia (>180 mg/dL) for both the virtual patients and the actual patients.

**Results:**

The subjects in the SH-VPP performed similarly vs. the actual patients (time in range: 78.1 ± 5.1% vs. 74.3 ± 8.1%, p = 0.11; time in hypoglycemia: 3.4 ± 1.3% vs. 2.8 ± 1.7%, p = 0.23). The subjects in the DH-VPP also performed similarly vs. the actual patients (time in range: 75.6 ± 5.5% vs. 71.9 ± 10.9%, p = 0.13; time in hypoglycemia: 0.9 ± 0.8% vs. 1.3 ± 1%, p = 0.19). While the VPPs tended to over-estimate the time in range relative to actual patients, the difference was not statistically significant.

**Conclusions:**

We have verified that a SH-VPP and a DH-VPP performed comparably with actual patients undergoing AP control using an identical control algorithm. The SH-VPP and DH-VPP may be used as a simulator for pre-evaluation of T1D control algorithms.

## Introduction

Mathematical models of the glucoregulatory system have been used within in-silico virtual patient simulations for many years [1, 2]. The FDA-approved UVA/Padova simulator, which was developed in 2008 (known as S2008 simulator), was one of the first simulators to model glucose-insulin metabolism. In the S2008 simulator, 100 virtual adults, 100 virtual adolescents, and 100 virtual children were generated by randomly drawing samples from the joint distribution of the parameters of the model [3]. At first, the 100 virtual adults were produced from a given nominal insulin sensitivity value and then the virtual children and adolescents were generated with higher and lower insulin sensitivity values. Since 2008, many studies have used the 2008 version of the UVA/Padova simulator for open loop [1, 4] and AP [5, 6] computer analyses. In 2013, due to hypoglycemia underestimation of the S2008 simulator, three new features were integrated. Dalla Man et al. [3] incorporated the non-linear effect of insulin action for glucose levels below a threshold. In addition, they added the glucagon kinetics and dynamics models to simulate the counter-regulatory behavior of glucagon for glucose levels below a threshold. They also modified the insulin-to-carb ratio as well as the correction factor for better representation of postprandial glucose excursions. The new simulator was named the S2013 simulator. Visentin et al. [7] validated the S2013 simulator with a database consisting of two sets of 24 glucose profiles recorded during one open loop study and one AP study across type 1 diabetes. Each glucose profile was controlled for 22 hours with two meal intakes. In both control trials, the variations of the meal intakes were negligible at each meal event across the patients. To validate the S2013 simulator, actual insulin profiles were given to the 100 virtual adults and the closest virtual adults were selected whose clinical outcomes were similar to the patients. Finally, the performance of the selected virtual adults were compared with the patients in terms of percent time spent in hyper- and hypoglycemia along with low and high blood glucose indices. They found better clinical consistency with the S2013, however unlike the S2008 simulator, the S2013 simulator overestimated the percent time spent in hypoglycemia significantly [7]. Later in 2016, to validate the S2013 simulator across type 1 diabetes and to better model time spent in hypoglycemia, Visentin et al. [8] fit the simulator to the actual dataset recorded from 47 people with T1D using a Bayesian approach. They found that the insulin sensitivity was around 30% less than the nominal values, showing that the insulin sensitivity of the S2013 simulator should be further modified to represent people with type 1 diabetes. While the S2013 simulator has been used by various research institutions to validate AP algorithms prior to running clinical studies, it is no longer commercially available and there is a need in the field for alternative VPPs to validate AP control algorithms. The Cambridge single-hormone simulator is another simulator developed for type 1 diabetes, which consists of 18 virtual patients [9]. The simulator was validated with a clinical dataset during overnight periods. In 2013, Haidar et al. [10] used the Cambridge simulator and developed a single-hormone virtual patient population by fitting the glucoregulatory model to glucose data of 12 young people with type 1 diabetes using a Markov chain Monte Carlo sampling method. In this paper, the glucoregulatory model used is similar to the Cambridge glucoregulatory model, except the insulin kinetics model is different. Our preliminary testing on the virtual populations showed that the insulin kinetics model published in Hovorka et al. [11] better reflects the physiological characteristics of adults with T1D.

The goal of this paper is to present two new open source VPPs that use statistical sampling to create an unlimited number of virtual patients. We present the mathematical model of both the dual-hormone VPP (DH-VPP) and the single-hormone VPP (SH-VPP). In the models, the most sensitive inter-subject parameters were statistically sampled to create the VPPs. The parameters associated with the insulin and glucagon sensitivity factors within the models were the parameters that were statistically sampled within the mathematical models. We describe how we validated the VPPs using glucose data, insulin data, and meal data collected from adults with type 1 diabetes during 3.5-day outpatient AP studies that involved self-selected meals, typical activities of daily living, and in-clinic aerobic exercise at 60% of the participant’s maximal VO_2_. We matched each virtual patient with one of the true patients from the AP study, matching them by their nearest TDIR and their weight. We then used the same control algorithm that was used in the AP outpatient studies [12–14] to control the glucose of the virtual patients under the identical meal scenarios that were given during the outpatient studies. We compared the clinical outcome measures from the outpatient study with those done on the VPP in-silico studies to validate the VPP. The SH-VPP and DH-VPP that are presented in this paper are made available through source code in Matlab as online supplementary material (S2 File) or by downloading the latest code from the Artificial Intelligence for Medical Systems (AIMS) lab GIT repository at https://github.com/petejacobs/T1D_VPP.

## Materials and methods

The SH-VPP and DH-VPP were generated based on glucoregulatory models consisting of insulin and glucagon kinetics and dynamics models and a glucose kinetics model. The SH-VPP was generated by statistically sampling the most sensitive inter-subject parameters of the insulin dynamics model. To generate the DH-VPP, the parameters of the insulin and glucagon dynamics models as well as one parameter in the glucose kinetics model were statistically sampled. Both VPPs were validated with experimental data.

### Glucoregulatory model

**Fig 1:**
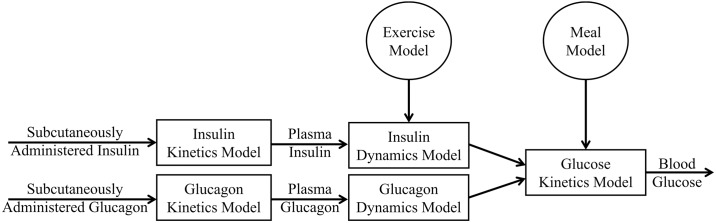
Block diagram of the glucoregulatory model.

The glucoregulatory models presented in this section have been previously published. The block diagram of the glucoregulatory model used in this study is shown in Fig 1. The single-hormone glucoregulatory model used in the SH-VPP is comprised of three main compartments: an insulin kinetics model, an insulin dynamics model and a glucose kinetics model. The DH-VPP is identical to the SH-VPP except that for the DH-VPP, two additional compartments were included: a glucagon kinetics and a glucagon dynamics model. Aerobic exercise can cause hypoglycemia in people with T1D [15] and it may be important for AP control algorithms to incorporate exercise detection and modified dosing to help avoid exercise-induced hypoglycemia [12, 16]. We have integrated an aerobic exercise model [17] into both the SH and DH-VPPs. Lastly, we have incorporated a meal absorption model into both VPPs.

The insulin kinetics model demonstrates the relationship between the subcutaneously administered insulin and plasma insulin concentration. In this study, we employed an insulin kinetics model developed by Hovorka et al. [11]. This model is outlined below:

$$\begin{array}{c} {\dot{S}}_{1}=u_{I}-\frac{S_{1}}{t_{max}}\\ {\dot{S}}_{2}=\frac{S_{1}}{t_{max}}-\frac{S_{2}}{t_{max}}\\ \dot{I}=\frac{S_{2}}{t_{max}V_{I}}-k_{e}I \end{array}$$ (1)

where S_1_ and S_2_ represent the masses of insulin [mU/kg] in two subcutaneous compartments, u_I_ represents the rate of insulin infusion [mU/kg/min], I represents the plasma insulin concentration [mU/L], and t_max_, V_I_ and k_e_ are the time-to-maximum absorption [min], distribution volume [L/kg] and elimination rate [min^-1^] of insulin. The insulin dynamics model, which describes the action of plasma insulin on glucose, was presented by Hovorka et al. [11]:

$$\begin{array}{c} \dot{X_{1}}=-k_{a1}X_{1}+S_{f1}k_{a1}I\\ \dot{X_{2}}=-k_{a2}X_{2}+S_{f2}k_{a2}I\\ \dot{X_{3}}=-k_{a3}X_{3}+S_{f3}k_{a3}I \end{array}$$ (2)

where X_1_ [min^-1^], X_2_ [min^-1^] and X_3_ [unitless] represent the effect of insulin on glucose distribution, disposal and suppression of Endogenous Glucose Production (EGP). S_f1_ [min^-1^ per mU/L], S_f2_ [min^-1^ per mU/L] and S_f3_ [per mU/L] are the insulin sensitivity factors and are the most sensitive inter-subject variables for describing variability in the glucoregulatory system of people with T1D. Selection of new insulin sensitivity factors enables us to generate new subjects within the VPPs. The variables k_a1_, k_a2_ and k_a3_ [min^-1^] are used as both appearance rates of insulin into the action compartments as well as the elimination rates of the insulin effects. The glucagon kinetics model, which represents the absorption rate of subcutaneously injected glucagon into plasma, was designed by Lv et al. (16):

$$\begin{array}{c} \dot{X_{1g}}=-\left(k_{1g}+k_{ge1}\right)X_{1g}+u_{g}\\ \dot{X_{2g}}=k_{1g}X_{1g}-k_{2g}X_{2g}\\ \dot{X_{3g}}=k_{2g}X_{2g}-k_{ge2}X_{3g} \end{array}$$ (3)

where X_1g_ and X_2g_ represent subcutaneous glucagon mass compartments and X_3g_ is plasma glucagon mass, all measured in mg/kg. u_g_ is the glucagon basal rate [mg/kg/min] infused from the glucagon pump. k_1g_ and k_2g_ are constant transfer rates [min^-1^]. k_ge1_ and k_ge2_ are elimination rates of glucagon from the inaccessible and accessible (plasma) compartments, respectively [min^-1^]. The glucagon dynamics model which describes the interaction between the plasma glucagon concentration and the EGP was previously described by Jacobs et al. [14]:

$$\begin{array}{c} \dot{Y}=\frac{10^{6}\times k_{c}\times S_{fGG}}{V_{dGG}}X_{3g}-k_{c}Y=k_{g}X_{3g}-k_{c}Y\\ Z=\dot{Y}\\ \dot{Z}=k_{g}k_{2g}X_{2g}-k_{g}k_{ge2}X_{3g}-k_{c}Z \end{array}$$ (4)

Y represents the effect of glucagon on EGP. We use the variable Z in Eq 4 to describe the glucagon model in [14] in state-space form. k_c_ is the clearance rate of glucagon from the remote compartment [min^-1^], S_fGG_ is the glucagon sensitivity factor [(ng/L)^-1^.min^-1^] and V_dGG_ is the glucagon volume of distribution [L/kg]. Similar to the insulin sensitivity factors, S_fGG_ is another sensitive inter-subject parameter and is used to generate the dual-hormone VPP. The glucose kinetics model, which estimates blood glucose with respect to insulin and glucagon actions and non-insulin mediated glucose uptake, was presented in Hovorka et al. [11] and Jacobs et al. [14]:

$$\begin{array}{c} \dot{Q_{1}}=-X_{1}Q_{1}-F_{01}^{c}-F_{R}+k_{12}Q_{2}+U_{G}+EGP_{0}\left(1-X_{3}+Y+k_{g3}Z\right)\\ \dot{Q_{2}}=X_{1}Q_{1}-k_{12}Q_{2}-X_{2}Q_{2} \end{array}$$ (5)

where Q_1_ and Q_2_ are the masses of glucose in the accessible (plasma) and non-accessible (rapidly-equilibrating interstitial) compartments, respectively [mmol/kg]. EGP_0_ is the basal endogenous glucose production at a theoretical zero insulin concentration [mmol/kg/min]. $F_{01}^{c}$ and F_R_ are the non-insulin mediated glucose uptake and the renal glucose clearance rate, respectively [mmol/kg/min]. For the SH-VPP, the Y and Z variables in Eq 5 are zero since no exogenous glucagon is considered to be given to the single-hormone virtual patient U_G_ represents the glucose absorption rate from meals [mmol/kg/min]:

$$U_{G}=\frac{D_{G}A_{G}\left(t-t_{0}\right)e^{-\frac{t-t_{0}}{t_{max,G}}}}{t_{max,G}^{2}}$$ (6)

where, t_max,G_ is the time-to-maximum appearance rate of glucose in Q_1_ [min], A_G_ is the carbohydrate bioavailability [unitless], t_0_ is the meal announcement time [min] and D_G_ is the estimated carbohydrate intake [mmol/kg]. Note that, for the in-silico simulations, D_G_ is converted from grams to mmol/kg to be compatible with the variables of the glucose kinetics model.

### Integration of exercise into the glucoregulatory model

Previously, we showed how an exercise model described by Hernandez-Ordonez et al. [17] could be incorporated into a VPP [14, 18, 19]. In the current paper, we include this exercise model in both the SH-VPP and DH-VPP and validate these populations relative to clinical data sets. We used the Hernandez et al. model to enable exercise to impact the peripheral insulin uptake, the peripheral glucose uptake, and the hepatic glucose production components of the model. Specifically, in the insulin dynamics model in Eq 2, the three insulin sensitivity factors (S_f1_, S_f2_ and S_f3_) are increased during the exercise bout as shown below in Eq 7.

$$\begin{array}{c} S_{f1-EX}=M_{PGU}M_{PIU}S_{f1}\\ S_{f2-EX}=M_{PGU}M_{PIU}S_{f2}\\ S_{f3-EX}=M_{HGP}S_{f3} \end{array}$$ (7)

where, M_PGU_ represents a percentage increment with respect to the basal peripheral glucose uptake (35 mg/min); M_PIU_ represents an increment of peripheral insulin uptake and M_HGP_ represents a percentage increment with respect to the basal hepatic glucose production (155 mg/min). These parameters are defined below:

$$\begin{array}{c} M_{PGU}=1+\frac{\Gamma_{PGUA}\times PAMM}{35}\\ M_{PIU}=1+2.4\times PAMM\\ M_{HGP}=1+\frac{\Gamma_{HGPA}\times PAMM}{155} \end{array}$$ (8)

where, PAMM represents the percentage of active muscular mass. In the testing described further below, the value of PAMM was set to 50% because the study participants were running on a treadmill with moderate intensity. Smaller values of PAMM (≈ 25%) were reported in [17, 20] for two-legged exercises. Γ_PGUA_ and Γ_HGPA_ are the glucose uptake and production from the active tissues respectively, and are assumed to have identical values during short-duration exercise according to Eq (9).

$$\overset{\cdot }{\Gamma_{PGUA}}=-\frac{1}{30}\Gamma_{PGUA}+\frac{1}{30}\Gamma_{\bar{PGUA}}$$ (9)

$\Gamma_{\overset{-}{PGUA}}$ represents the peripheral glucose uptake by active tissue in steady state and is a function of PVO_2max_ according to the following equation:

$$\Gamma_{\bar{PGUA}}=0.006{\left(PVO_{2max}\right)}^{2}+1.2264\left(PVO_{2max}\right)-10.1958$$ (10)

where, PVO_2max_ is the percentage of the maximum oxygen consumption during exercise. It was calculated using the metabolic equivalents (MET) as shown below:

$$PVO_{2max}=\frac{MET}{MET_{max}}$$ (11)

where MET_max_ is the maximum energy expenditure estimated during a VO_2max_ test. The values of MET and MET_max_ were estimated in the clinical evaluations described further below with the heart rate and accelerometry data recorded by a Zephyrlife BioPatch. The MET estimation was further personalized by incorporating anthropometric characteristics of each individual [21]. During non-exercise periods, M_PGU_, M_PIU_ and M_HGP_ are close to one, and insulin sensitivity factors do not change.

### Clinical data

Real-world meal scenarios (Table A in S1 File) were used from 20 patients with T1D who underwent two separate 3.5-day randomized outpatient AP trials. In one trial, glucose levels were controlled with insulin and in the other, glucose levels were controlled with both insulin and glucagon. Subjects were enrolled at the Oregon Health and Science University and the control algorithm used was the OHSU-FMPD controller [13, 16]. Participants in the study spent the first and fourth day of the study at the hospital eating known meals and participating in aerobic exercise at 60% of their maximal VO_2_. The in-clinic exercise bouts lasted for 45 minutes and were performed 2 hours after lunch. Table 1 summarizes the characteristics of the participants of the study. For more information about the study and participants, refer to Castle et al. [12] and clinicaltrials.gov (Clinical trial reg. no. NCT02862730).

**Table 1:** Baseline characteristics of the participants in the AP study [12].

		Value	Range
**Age** (years)		35.0 ± 4.7	27–45
**Sex**	Female	14 (70%)	
	Male	6 (30%)	
**Weight** (kg)		76.3 ± 14.6	55.6–104.7
**Height** (cm)		172.0 ± 10.1	156.0–189.0
**HbA**_**1c**_ (%)		7.6 ± 0.8	6.0–9.1
**TDIR** (units)		42.3 ± 16.0	18.0–93.0
**Duration of Diabetes** (year)		20.2 ± 8.0	8.0–37.0
**Maximum HR**_**Ex**_ (bpm)		182.4 ± 9.0	170.0–199.0
**Maximum MET**_**Ex**_		11.7 ± 2.5	6.5–16.5

HR_Ex_: heart rate during exercise; MET_Ex_: metabolic equivalent during exercise. Data is reported as mean ± standard deviation.

### Single-hormone VPP

A Single-hormone VPP was generated for running single-hormone simulations. The nominal values given for S_f1_, S_f2_, and S_f3_ from Eq (2) were derived for people without T1D [11]. We updated S_f1_, S_f2_ and S_f3_ to represent the sensitivity of insulin for people with T1D. TDIR was used to personalize insulin sensitivity for each virtual patient. TDIR is the amount of insulin required by a person with diabetes during 24 hours. Insulin sensitivity is inversely proportional to TDIR. Insulin sensitivity is a measure of how sensitive the body is to insulin. Generally, subjects with higher weight have higher TDIR because a larger body oftentimes requires more insulin. To consider a range of TDIR values that relate to different insulin sensitivities, we created a sensitivity composite (Sc) that ranged from 0.1 to 2; this sensitivity composite was multiplied by the nominal values of S_f1_, S_f2_ and S_f3_ in Eq (2) to generate a range of basal insulin values. Basal insulin (*I*_*basal*_) for each sensitivity composite was determined through simulations where the basal insulin rates at each value of Sc yielded a steady state glucose level of 115 mg/dl. The units of *I*_*basal*_ is mU/kg/min, which is also shown in Eq 1. To convert the units of *I*_*basal*_ to U/hr., we multiplied *I*_*basal*_ by a fixed weight of 76.3 kg obtained from the average weight across a clinical dataset of people with T1D, shown in Table 1. The daily basal requirement was then computed as *I*_*basal*_ × 24. This total daily basal insulin requirement (basal-TDIR) did not include insulin given for meals. The TDIR, which includes meals and basal insulin, was estimated by multiplying the TDIR by a factor of 1.8 which was empirically validated on an OHSU clinical dataset across people with T1D, implying that our VPP obtained 44.4% of their daily insulin from meals and 55.6% of their daily insulin from basal. Walsh *et al*. [22] introduced a similar impact of basal-TDIR on TDIR. They showed that the basal-TDIR is approximately 48% of the TDIR.

**Fig 2:**
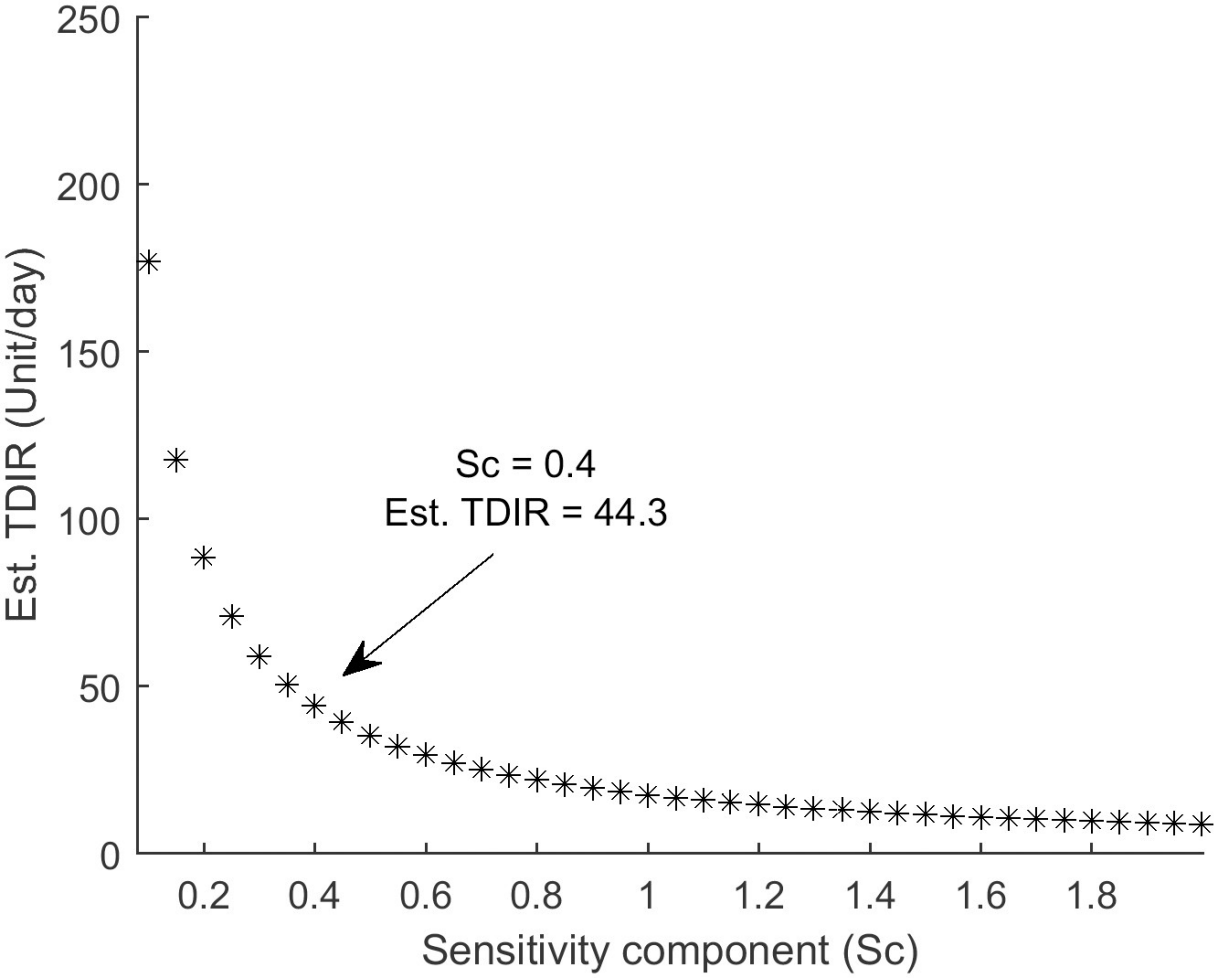
Estimated TDIR across Sc values. Sc of 0.4 was selected as the insulin sensitivity modifier for people with T1D for generating VPPs of people with an average weight of 76.3 kg.

Fig 2 shows the relationship between the Sc value and the TDIR. Based on the mean TDIR from a clinical dataset of approximately 45 units/day, an Sc of 0.4 was chosen as the insulin sensitivity modifier across subjects with T1D. Selection of an Sc of 0.4, results in a corresponding reduction of insulin sensitivity of people in our VPP such that they have a 60% lower insulin sensitivity than people without T1D. A similar relationship between the insulin sensitivity of people with and without T1D was investigated by Rickels *et al*. [23] in a euglycemic clamp study. It is important to note that this Sc of 0.4 for generating a large VPP was determined using an average weight of 76.3 kg. obtained from a clinical data set of people with T1D. Under the section “Validating VPPs under real-world meal scenarios” we will show how to generate Sc values for individual patients with specific weights.

Next, virtual patients with T1D were created by statistically sampling from the distributions of the updated insulin sensitivity factors given an ad-hoc 75% correlation between S_f1_ and S_f2_, and 25% correlation between S_f2_ and S_f3_. In addition, the weight of the virtual patients was sampled from a normal distribution, with mean of 76.3 kg and standard deviation of 14.6 kg that was obtained based on the clinical data described further above.

After sampling the parameters of each virtual patient, the physiologic feasibility of each virtual patient was evaluated through two tests:

1. Steady-state glucose levels of each virtual patient in the absence of insulin should exceed 300 mg/dl.
2. Delivery of high-dose insulin (15 unit/hr) to each virtual patient should result in a low steady-state glucose level (typically less than 100 mg/dl from the baseline steady-state glucose).

**Fig 3:**
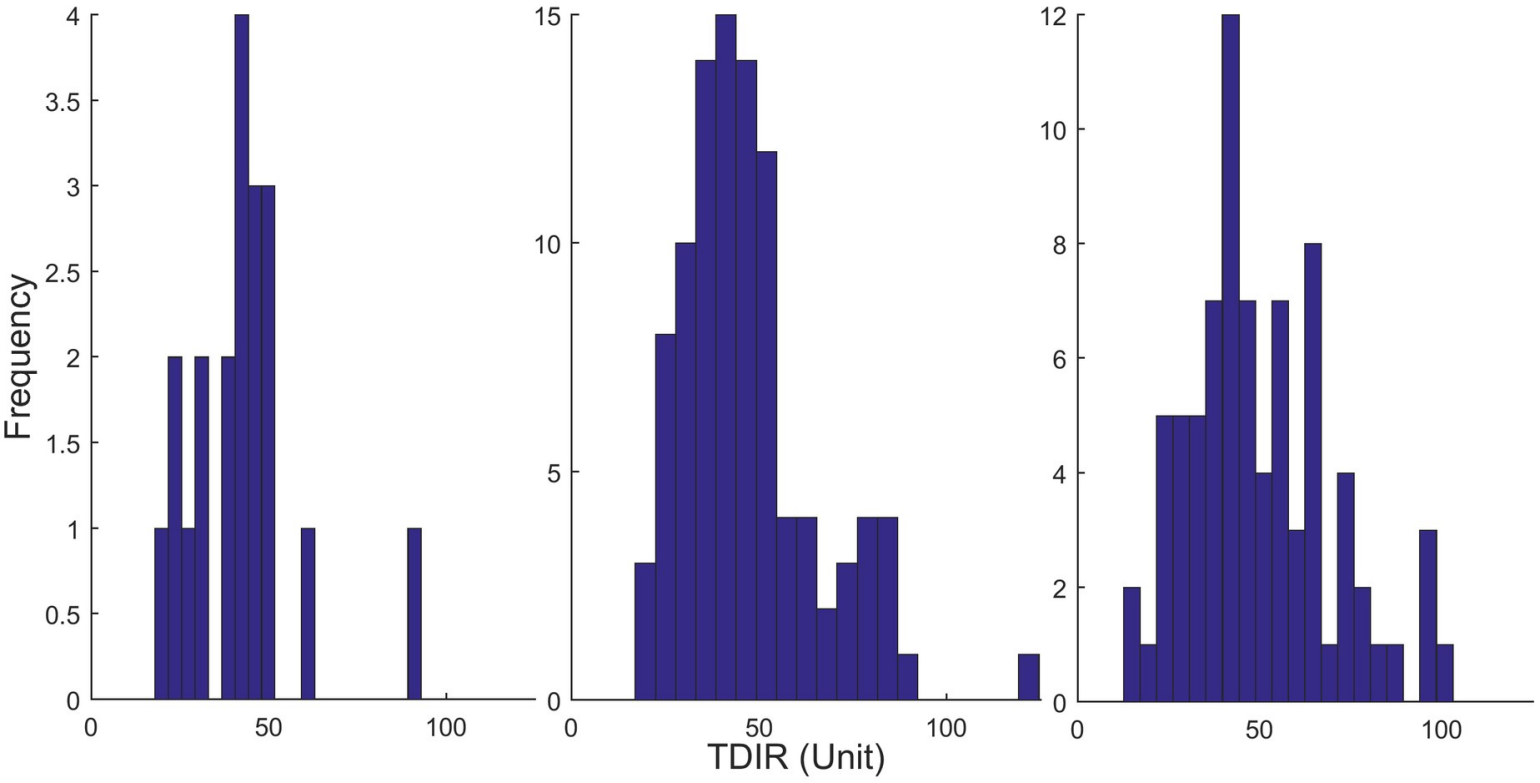
Histogram of the TDIR values of the clinical patients (left), SH-VPP (middle) and DH-VPP (right).

A total of 99 virtual individuals out of 100 passed the above criteria. Fig 3 shows the histogram of the TDIR values of the single-hormone VPP.

### Dual-hormone VPP

For generating DH-VPP, we first followed the instructions of generating single-hormone VPP and reduced insulin sensitivity factors (S_f1_, S_f2_ and S_f3_) by 60%. Then, we changed the most sensitive inter-subject parameters (EGP_0_, S_f1_, S_f2_ and S_f3_, S_fGG_, k_c_ and kg_3_) of the glucoregulatory model across each subject. Similar to the single-hormone VPP, we assumed a normal distribution of these parameters and we randomly sampled from these distributions to create a new virtual patient. To determine the physiologic feasibility of the randomly drawn parameters, each parameter set was required to pass four clinically-relevant criteria, listed below.

1. Steady-state glucose levels in each virtual patient in the absence of insulin should exceeds 300 mg/dl.
2. Delivery of high-dose insulin (15 U/hr) to each virtual patient should result in a low steady-state glucose level (typically less than 100 mg/dl from the baseline steady-state glucose).
3. Delivery of high-dose glucagon (20 mcg/kg/hr) to each virtual patient should result in a significant rise in glucose within 2 hours of the dose, greater than 50 mg/dl above the baseline steady-state glucose [18].
4. Delivery of a small dose of glucagon (0.2 mcg/kg/hr) to each virtual patient should not result in a response greater than 100 mg/dl above baseline steady-state glucose [18].

A total of 90 out of 100 virtual patients passed the below criteria and were selected for the dual-hormone VPP. Fig 3 shows the histogram of the TDIR values of the DH-VPP. Table 2 shows all the numerical values of the parameters of the glucoregulatory models. The parameters that were statistically sampled to create the virtual patient populations are shown along with their standard deviations.

**Table 2:** The numerical values of the parameters of the glucoregulatory models.

Parameters	Values	Parameters	Values	Parameters	Values	Parameters	Values
$F_{01}^{c}$	0.0097	V_G_	0.16	k_12_	0.066	t_max,G_	40
t_max_	55	k_e_	0.138	V_I_	0.12	A_G_	0.8
k_a1_	0.006	k_a2_	0.06	k_a3_	0.03	k_1g_	0.0065
k_ge1_	0.0772	k_ge2_	0.0357	V_dGG_	0.19	k_ge1_	0.0772
S_f1_ (× 10^−4^)	21 ± 5.9	S_f2_ (× 10^−4^)	3.5 ± 1.4	S_f3_ (× 10^−4^)	214 ± 5.9	EGP_0_ (× 10^−2^)	1.61 ± 0.15
S_fGG_ (× 10^−2^)	1.7 ± 0.47	k_c_ (× 10^−2^)	6.0 ± 1.95	k_g3_	140 ± 39.9	k_2g_	0.02777

Data is shown as the mean and standard deviation for the variable parameters.

For the SH-VPP, only S_f1_, S_f2_, S_f3_ were sampled across the virtual subjects.

For the DH-VPP, only S_f1_, S_f2_, S_f3_, S_fGG_, k_c_, k_g3_ and EGP_0_ were sampled across the virtual subjects.

### Validating VPPs under real-world meal scenarios

To validate the VPPs, we matched clinical patients with T1D with their virtual twin from the virtual patient population. In this section, we describe how we matched real-world patients with T1D with their virtual twin. The 99 single-hormone virtual patients and 90 dual-hormone virtual patients described above are the patients that should be used typically to run simulations on a glucose control algorithm. For the purpose of validation, we generated 20 new virtual patients that were created to match actual patients with T1D by weight and TDIR. The only difference between the methods described above and those used to match the clinical patients with their virtual twin was the determination of Sc values. Unlike the methods described above whereby the Sc vs. TDIR relationship (Fig 2) was generated using the *average weight* of patients (76.3 kg from Table 1), the Sc vs. TDIR for the validation data was generated individually for each clinical patient using their *actual weight*. Meal scenarios describing daily meal content and pattern of consumption were acquired from a previous clinical study assessing single hormone and dual hormone artificial pancreas technologies [12]. Twenty 3.5-day meal scenarios from the single-hormone clinical trial and twenty 3.5-day meal scenarios from the dual-hormone clinical trial were collected and used to deliver to the virtual patient population (Table A in S1 File). Virtual patients were matched to clinical study participants by closest match of TDIR and weight. Matching a virtual patient to a study participant was done by first creating a TDIR vs. sensitivity component (Sc) graph like the one shown in Fig 2 using the participant’s actual weight. The Sc that most closely corresponded to a given participant’s TDIR was determined and a temporary set of 100 virtual patients was generated using the methods described above under the sections Single-hormone VPP and Dual-hormone VPP. Then, the TDIR of each of the temporary virtual patients was compared to the participant’s TDIR and finally the desired virtual patient whose TDIR was the closest was identified. By using this approach, we ensured that both weight and TDIR of each actual patient were used to identify the closest virtual patient. This approach was repeated for all 20 actual patients from each clinical study trial and the 20 closest virtual twins were identified. We then used the same OHSU-FMPD control algorithm that was used in the outpatient AP studies to control the glucose levels of each of the 20 virtual patients under the dual-hormone and single-hormone meal scenarios. The control algorithm was implemented in Java and has been previously described [13, 14]. The glucose profiles of the virtual patients were compared with the related actual glucose profiles controlled by the same controller during the in-vivo trial. For the in-silico simulations, the system was further challenged by introducing a randomly selected -30% to 30% meal uncertainty applied to each carbohydrate intake at each meal scenario. Since insulin is known to vary during the day [24], circadian variability of insulin sensitivity was introduced to the insulin sensitivity parameters (S_f1_, S_f2_ and S_f3_) within each virtual patient by varying these parameters with respect to time of day using Eq (12):

$${\text{S}}_{\text{fi}}\left(\text{t}\right)={\text{S}}_{\text{fi}}^{*}\times (1+0.3\text{sin}\left(\frac{2\pi }{24\times 60/\text{Ts}}\times \text{t}+2\pi \times \text{RND}\right),\;\text{i}=1,2,3$$ (12)

where, RND is a random variable generated from a uniform distribution between 0 and 1; Ts is the sampling interval (5 minutes). S_fi_* denotes the nominal value of each of the insulin sensitivity factors. The phase of the circadian insulin sensitivity was randomly initialized at the start of the study using the RND command, and this phase was fixed for all virtual patients. This approach helped us to compare the performance of all virtual patients similarly as the phase shift remained constant. We additionally modeled glucose sensor noise using the glucose sensor noise model described by Facchinetti et al. [25, 26]. During this study, meal scenarios and exercise sessions were imposed on the virtual subjects as determined by the existing study data. And as described under the section ‘Integration of exercise into the glucoregulatory model’, the nominal insulin sensitivity (S_f_*) in Eq 12 is changed during exercise according to Eq 7. It is increased during the exercise period and returned to the original value at the end of exercise.

### Evaluation metrics and statistical analysis

We assessed accuracy of the VPP by comparing the primary outcome measures of the VPP with primary outcome measures acquired during the clinical study. The primary outcome measures for the validation of VPPs included the percent time in hypoglycemia (<70mg/dl) and the percent time in target range (70–180 mg/dl). The secondary outcome measures for validation of the VPPs included the percent time in hyperglycemia (>180mg/dl) and the low and high blood glucose indices [27]. We report errors in the clinical outcome metrics (e.g. time in range, time in hypoglycemia, time in hyperglycemia) as mean absolute error (MAE) whereby error in the VPP outcome metrics are calculated relative to the outcome metrics obtained from the clinical study. To assess the statistical difference between the simulated and the actual glucose profiles, the student t-test was used, with significance level set to 5%.

$$MAE=\frac{1}{M}\sum_{i=1}^{M}\left|Outcome_{clinical}-Outcome_{simulation}\right|$$ (13)

Where M is the number of meal scenarios used in the validation step of the VPPs. The MAE was computed for each of the outcome metrics.

## Results

Fig 3 show the histogram of the patients, SH-VPP and DH-VPP. The range of the TDIR values of SH-VPP started at 20 units and ended at 120 units. The peak of the histogram was around 40–45 units showing that the TDIR values of the SH-VPP were well-scaled regarding the average TDIR value shown in Table 1. A similar range is also observable in DH-VPP. The peak of the histogram occurred for TDIR values between 40–45 units, however the minimum TDIR spanned to smaller levels for several virtual patients simulating the situations where certain individuals with T1D may require less insulin.

**Fig 4:**
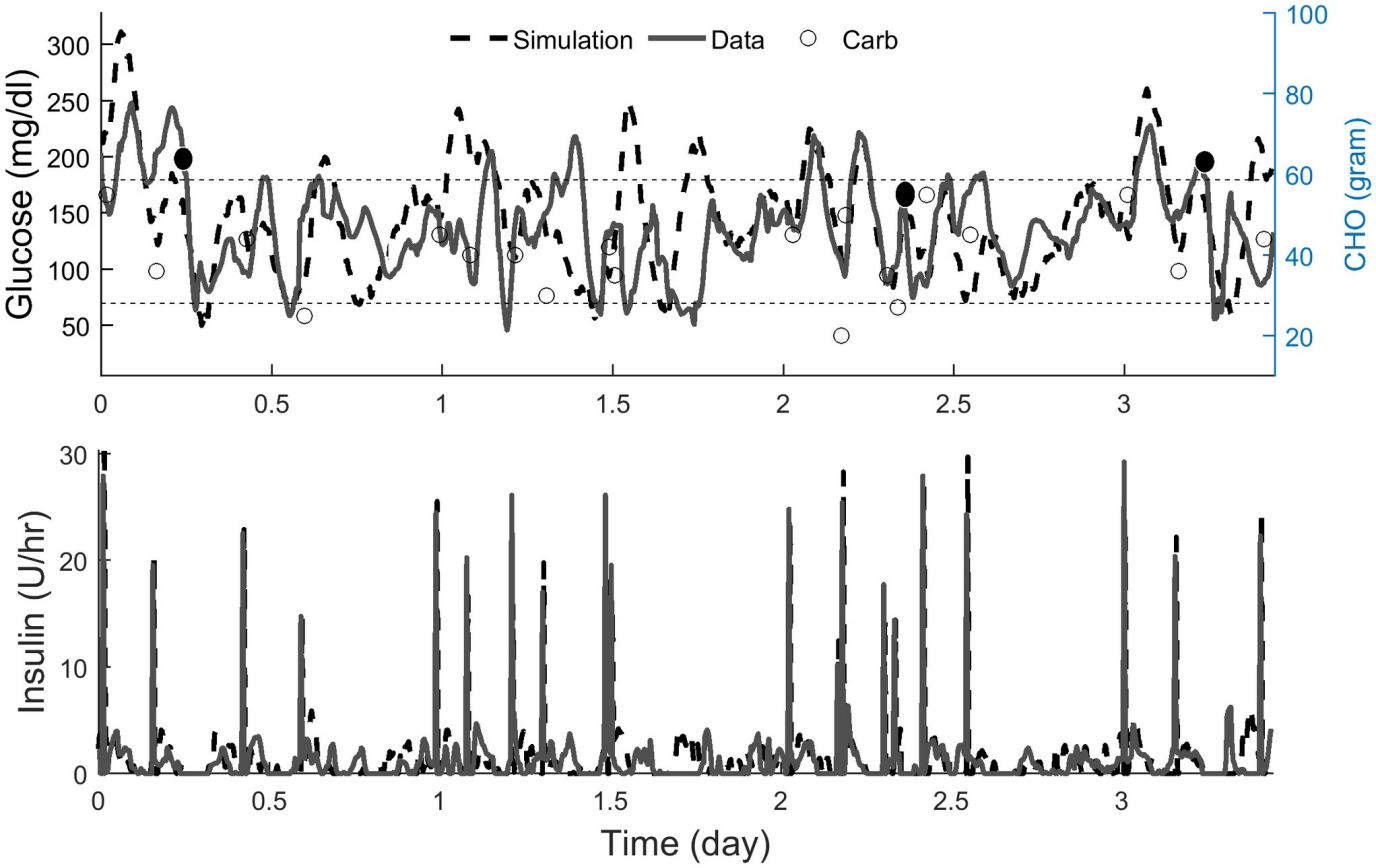
Simulated vs. actual glucose and insulin profiles of one representative subject in single-hormone trial. Both experiments were initialized at 8:00 am. Carbs are shown with circles. Filled circles show the start of exercise. Higher resolution data from this study is shown in Fig A in S1 File.

**Fig 5:**
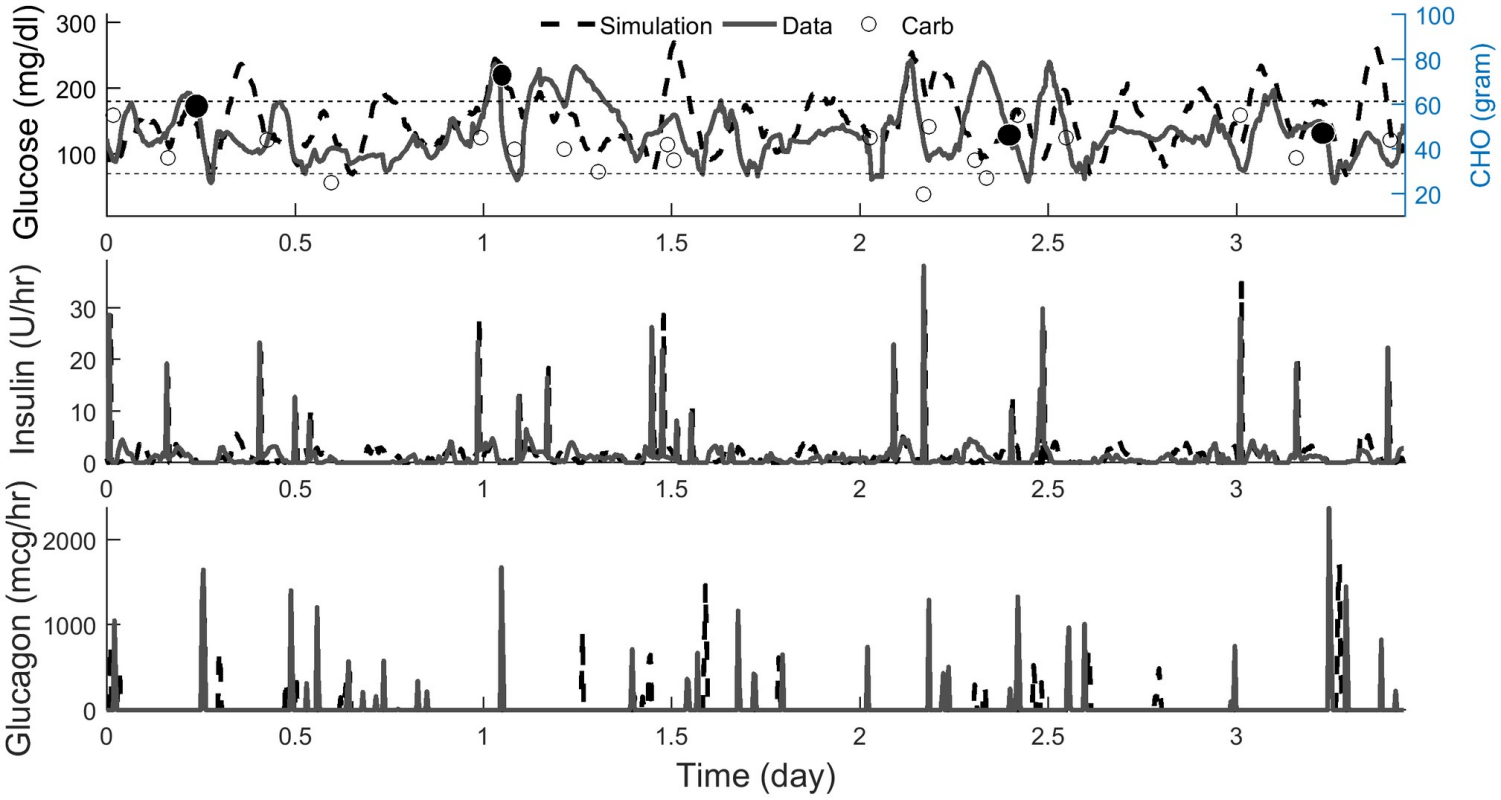
Simulated vs. actual glucose, insulin and glucagon profiles of one representative subject in dual-hormone trial. Both experiments were initialized at 8:00 am. Carbs are shown with circles. Filled circles show the start of exercise. Higher resolution data from this study is shown in Fig B in S1 File.

Figs 4 and 5 show the comparison between the simulated and the actual glucose profile for one representative subject in SH and DH trials. Overall, the dynamic responses of the simulated glucose profiles during meal events and exercise bouts were similar to the actual one. Higher resolution figures comparing the simulations with clinical data are provided in Figs A and B in S1 File.

Tables 3 and 4 show the clinical study outcomes in comparison with the in-silico control simulation outcomes of the VPPs. Table 3 shows results from the single-hormone study and Table 4 shows results from the dual-hormone study. The tables also show the statistical analysis of each outcome and a p-value indicating whether the VPP outcome was statistically different than the clinical outcome using a two-tailed t-test analysis. For all outcome measures, the SH-VPP was not statistically different than the true population. The time spent in hyperglycemia was slightly underestimated by the SH-VPP, which was not significant but was trending towards significant (*p* = .08). The MAE of time spent in hyperglycemia was high for SH-VPP showing that the meal model should be further improved to better represent glucose levels above 180 mg/dl. The outcome measures for the DH-VPP during the in-silico AP simulation are not statistically different than the outcome measures for the actual patients during the AP clinical study as shown in Table 4, however the HBGI is trending towards being significant (*p* = .06).

**Table 3:** Outcome metrics of the single-hormone VPP across the selected virtual patients.

Single Hormone VPP	Clinical Results	Simulated Results	*p*-value	MAE (%)
**Time in hypoglycemia** (%)	2.8 ± 1.7	3.4 ± 1.3	0.23	2
**Time in hyperglycemia** (%)	22.9 ± 8.8	18.4 ± 5.3	0.08	9.6
**Time in range** (%)	74.3 ± 8.1	78.1 ± 5.1	0.11	8.4
**LBGI**	3.1 ± 1	3.5 ± 0.9	0.24	1.2
**HBGI**	6.2 ± 1.7	5.9 ± 1.2	0.47	1.9

**Table 4:** Outcome metrics of the dual-hormone VPP across the selected virtual patients.

Dual Hormone VPP	Clinical Results	Simulated Results	*p*-value	MAE (%)
**Time in hypoglycemia** (%)	1.3 ± 1	0.9 ± 0.8	0.19	1.1
**Time in hyperglycemia** (%)	26.7 ± 11.4	23.5 ± 5.7	0.21	8.8
**Time in range** (%)	71.9 ± 10.9	75.6 ± 5.5	0.13	8.3
**LBGI**	2.3 ± 1.3	1.9 ± 0.8	0.30	1.3
**HBGI**	7.2 ± 2.3	6.2 ± 1.1	0.06	1.9

## Discussion and conclusion

In this paper, we described the design of two T1D virtual patient populations that can be used to evaluate single-hormone and dual-hormone control algorithms within automated drug delivery systems for helping people with T1D better manage their glucose levels. These virtual populations were validated against clinical data acquired from real-world patients with T1D [12]. The results showed no significant difference between the performance outcome measures of the VPPs and the true patients when treated with an automated control algorithm intervention and when given identical meals. In this study, we were able to validate the VPP on a clinical data set whereby patients with T1D were matched with their in-silico virtual twin by TDIR and weight. Both real patients and virtual patients were given the same meals and exercise regimen while their glucose was controlled using the same control algorithm. It is important to emphasize that, while we used just one control algorithm to validate the VPPs, this does not mean that these VPPs are compatible with just a single control algorithm. Any control algorithm can now be used with the VPPs. We have simply used a single control algorithm to validate that when virtual patients and actual patients are given comparable amounts of insulin, glucagon, meals, and exercise, the glycemic outcome metrics between the virtual and actual patients are not statistically different.

For further evaluating the VPPs, we compared the performance of the SH-VPP with the free version of the single-hormone UVA/Padova simulator. In this comparison, we only used 10 of the real-world meal scenarios because the UVA/Padova simulator deletes meal events that occur within 30 minutes of a prior meal. For the purpose of comparison, we eliminated 10 of the 20 meals, which had meal events occurring within 30 minutes of each other. For each of the 10 selected meal scenarios, a relevant UVA/Padova virtual subject was identified based on weight and TDIR, similar to the selected virtual patients descried above. Because the single-hormone UVA/Padova simulator does not have an exercise model, we could only compare the performance of the UVA simulator with the VPP population at the second day, when no exercise took place in the study. Table 5 shows the comparison between the single-hormone VPP, the single-hormone UVA/Padova simulator and the clinical data across the 10 selected meal scenarios. Both simulators agreed closely on average with the clinical data for the time in range outcome measure and time in hyperglycemia. However, they misestimated the time in hypoglycemia compared to the clinical data. The MAE of the UVA simulator relative to the clinical data for time in range was 33.4%, which was significantly higher than the MAE of the SH-VPP, which was 15.8%. The MAE for the percent time in hypoglycemia of the UVA/Padova simulator relative to the clinical data was comparable with the SH-VPP showing slightly higher error (0.76% for UVA/Padova vs. 1.7% for SH-VPP).

**Table 5:** Comparison between the simulators and the clinical data across the 10 selected meal scenarios.

Simulators/outcomes	Time in hypoglycemia (%)	Time in hyperglycemia (%)	Time in range (%)
**Clinical data**	0.77	24.72	74.51
**SH-VPP**	2 (0.01)	15.4 (0.11)	82.6 (0.16)
**UVA/Padova**	0 (0.02)	30.4 (0.62)	69.6 (0.67)

Data is shown as the mean, and the *p*-value in parenthesis for the comparison.

While the SH-VPP and the DH-VPP on average resulted in a good match with the clinical data, the MAE was higher than we would prefer for the percent time in range and the percent time in hyperglycemia. This indicates that for certain individuals, there was not always a good match between the in-silico model and the weight/TDIR matched clinical participant. There are several reasons why this was the case. First, for the clinical study we did not know the true meal amount consumed by the patient and instead could only estimate based on their input during the clinical study. This is why we imposed a +/- 30% variability in the carbohydrate consumed by the virtual patients at each meal. This meal estimation uncertainty will inevitably cause error between the participant and the in-silico matched patient. Second, there was uncertainty of the time when clinical study participants delivered their rescue carbs for times when their glucose dropped below 70 mg/dl. In our simulations, the virtual patient was given a rescue carbohydrate 10 minutes after glucose dropped below 70 mg/dl. For the clinical participant, the rescue carbohydrate delivery could have been given at a different time, which would contribute to error. Third, it is known that insulin sensitivity can vary throughout the day. We modeled this insulin sensitivity variability by varying each virtual patient’s insulin sensitivity by +/- 30% throughout the day. This potentially inaccurate estimation of circadian insulin sensitivity variability could further explain the error observed. Fourth, the exercise model that we used in the VPP was validated on continuous and non-intermittent aerobic exercise with constant PVO_2_ [17]. We further assumed in the exercise model that the PAMM was 50% for all subjects. However, we know that there was some variability in the exertion of the subjects throughout the exercise sessions and it is probable that the PAMM for all of the subjects was not exactly 50%. This would have been a cause for further error observed. Palumbo et al. [28] describe how PVO_2max_ can be adapted to a patients’ specific physiology and adapt based on duration and intensity of exercise. In the future, we will need to do a similar type of adaptation to better model the impact of exercise duration, type, and intensity on glycemic control. A further reason for differences between the VPPs and the clinical subjects was that the VPP used a model of the CGM noise that was derived using the Dexcom G4 glucose sensor, whereas the data collected from the clinical study was done using the Dexcom G5 sensor. Despite these various factors that contributed to individual differences between the virtual patients and the clinical study participants, we remain confident that on average the SH-VPP and DH-VPP are sufficiently accurate for use in designing and evaluating AP control algorithms prior to an actual clinical study. The average outcome measures from the clinical study were not statistically significantly different than those of the in-silico study. And the MAE was lower than other stimulators that have been used in the past to evaluate AP control algorithms prior to in-vivo studies. In the future, we plan to leverage the clinical data set to try to improve our models by using system identification approaches such as Markov Chain Monte Carlo (MCMC) approaches. While the goal of the current work was to use the clinical data to estimate the accuracy of the VPP, we can certainly try to achieve a closer match to the clinical data by identifying each individual’s insulin sensitivity, carbohydrate sensitivity, and exercise model parameters.

In conclusion, two new single and dual-hormone VPPs were presented and validated against a clinical data set. On average, there was not a significant difference in outcome measures between the clinical data and the in-silico data, indicating that both VPPs may be used for pre-clinical evaluation of AP algorithms.

## Supporting information

S1 File (DOCX)

S2 File (7Z)
