# Supplementary material for: A statistical virtual patient population for the glucoregulatory system in type 1 diabetes with integrated exercise model
Source: PLoS One. 2019 Jul 25;14(7):e0217301. doi: 10.1371/journal.pone.0217301 (PMC6657828; doi:10.1371/journal.pone.0217301)
Supplement: S1 File — (DOCX) [file pone.0217301.s001.docx]

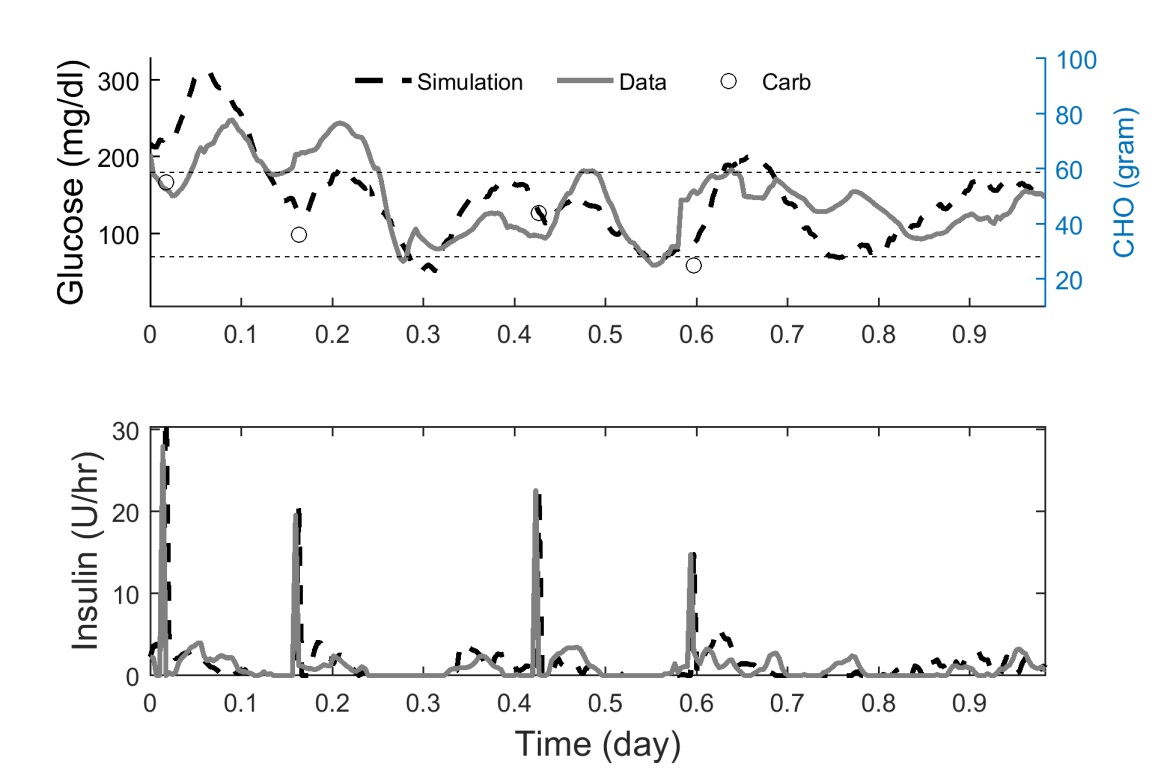


**Fig A. Simulated vs. actual glucose and insulin profiles of the representative subject shown in Figure 4 for one-day simulation**. **Both experiments were initialized at 8:00 am. Carbs are shown with circles.**


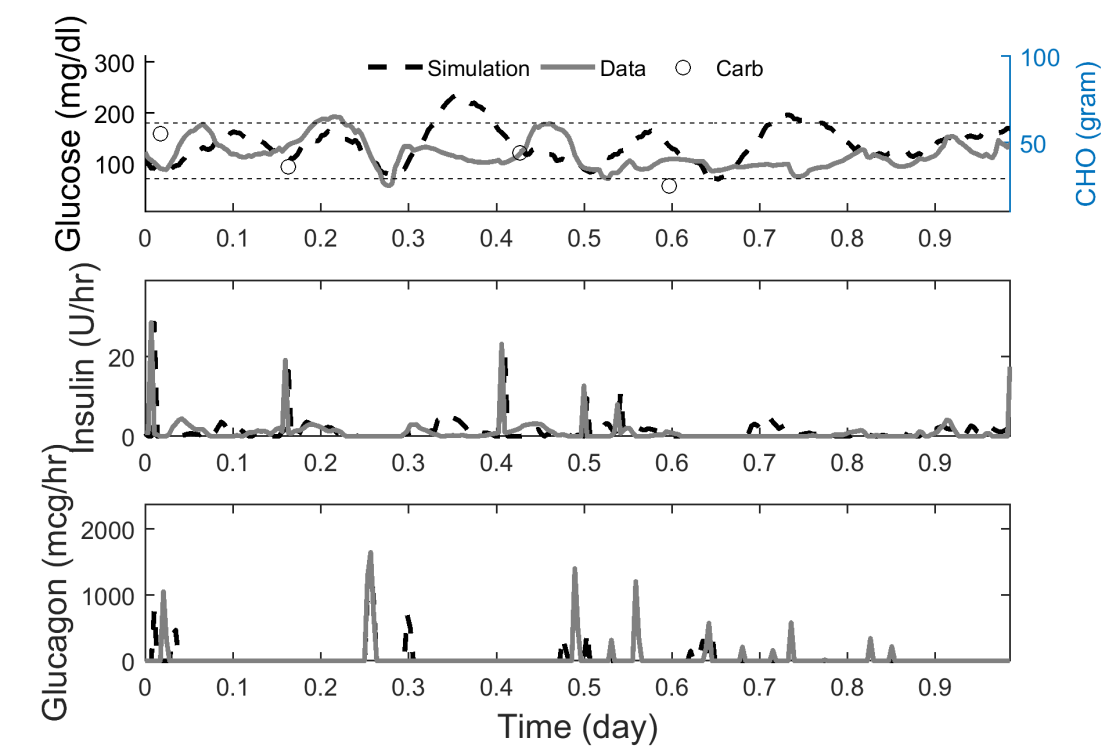


**Fig B. Simulated vs. actual glucose, insulin and glucagon profiles of the representative subject shown in Figure 5 for one-day simulation**. **Both experiments were initialized at 8:00 am. Carbs are shown with circle.**

Table A: Information of the meal scenarios

| Meal Scenario | Mean carbs and std |
| --- | --- |
| 1 | 40.2 ± 9.9 |
| 2 | 72.8 ± 36.6 |
| 3 | 45.1 ± 8.7 |
| 4 | 42.6 ± 30.1 |
| 5 | 42.8 ± 28.7 |
| 6 | 47.4 ± 21.9 |
| 7 | 46.4 ± 15.5 |
| 8 | 32.6 ± 18.9 |
| 9 | 40.2 ± 34.7 |
| 10 | 38.9 ± 20 |
| 11 | 45.1 ± 25.4 |
| 12 | 31.6 ± 16.9 |
| 13 | 40.4 ± 30.4 |
| 14 | 55.8 ± 35.2 |
| 15 | 57.2 ± 15.9 |
| 16 | 33.9 ± 21.6 |
| 17 | 48.4 ± 24.8 |
| 18 | 32.2 ± 11.1 |
| 19 | 40.9 ± 21.7 |
| 20 | 38.9 ± 2.6 |
| Average | 43.7 ± 9.7 |
